# CAG repeat length in the androgen receptor gene is related to age at diagnosis of prostate cancer and response to endocrine therapy, but not to prostate cancer risk

**DOI:** 10.1038/sj.bjc.6690746

**Published:** 1999-10

**Authors:** O Bratt, Å Borg, U Kristoffersson, R Lundgren, Q-X Zhang, H Olsson

**Affiliations:** Departments of Urology, Oncology, Clinical Genetics, University of Lund, Lund, SE-221 85, Sweden

**Keywords:** prostatic neoplasms, cancer risk, androgen receptor, genetics, epidemiology

## Abstract

The length of the polymorphic CAG repeat in the N-terminal of the androgen receptor (AR) gene is inversely correlated with the transactivation function of the AR. Some studies have indicated that short CAG repeats are related to higher risk of prostate cancer. We performed a case–control study to investigate relations between CAG repeat length and prostate cancer risk, tumour grade, tumour stage, age at diagnosis and response to endocrine therapy. The study included 190 AR alleles from prostate cancer patients and 186 AR alleles from female control subjects. All were whites from southern Sweden. The frequency distribution of CAG repeat length was strikingly similar for cases and controls, and no significant correlation between CAG repeat length and prostate cancer risk was detected. However, for men with non-hereditary prostate cancer (*n* = 160), shorter CAG repeats correlated with younger age at diagnosis (*P* = 0.03). There were also trends toward associations between short CAG repeats and high grade (*P* = 0.07) and high stage (*P* = 0.07) disease. Furthermore, we found that patients with long CAG repeats responded better to endocrine therapy, even after adjusting for pretreatment level of prostate-specific antigen and tumour grade and stage (*P* = 0.05). We conclude that short CAG repeats in the AR gene correlate with young age at diagnosis of prostate cancer, but not with higher risk of the disease. Selection of patients with early onset prostate cancer in case–control studies could therefore lead to an over-estimation of the risk of prostate cancer for men with short CAG repeats. An association between long CAG repeats and good response to endocrine therapy was also found, but the mechanism and clinical relevance are unclear. © 1999 Cancer Research Campaign


*British Journal of Cancer* (1999) **81**(4), 672–676
© 1999 Cancer Research Campaign
Article no. bjoc.1999.0746

### CAG repeat length in the androgen receptor gene is related to age at diagnosis of prostate cancer and response to endocrine therapy, but not to prostate cancer risk

**O Bratt[1], Å Borg[2], U Kristoffersson[3], R Lundgren[1], Q-X Zhang[2] and H Olsson[2]**

Departments of [1]Urology, [2]Oncology and [3]Clinical Genetics, University of Lund, SE-221 85 Lund, Sweden

**Summary** The length of the polymorphic CAG repeat in the N-terminal of the androgen receptor (AR) gene is inversely correlated with the transactivation function of the AR. Some studies have indicated that short CAG repeats are related to higher risk of prostate cancer. We performed a case–control study to investigate relations between CAG repeat length and prostate cancer risk, tumour grade, tumour stage, age at diagnosis and response to endocrine therapy. The study included 190 AR alleles from prostate cancer patients and 186 AR alleles from female control subjects. All were whites from southern Sweden. The frequency distribution of CAG repeat length was strikingly similar for cases and controls, and no significant correlation between CAG repeat length and prostate cancer risk was detected. However, for men with non-hereditary prostate cancer ($n$ = 160), shorter CAG repeats correlated with younger age at diagnosis ($P$ = 0.03). There were also trends toward associations between short CAG repeats and high grade ($P$ = 0.07) and high stage ($P$ = 0.07) disease. Furthermore, we found that patients with long CAG repeats responded better to endocrine therapy, even after adjusting for pretreatment level of prostate-specific antigen and tumour grade and stage ($P$ = 0.05). We conclude that short CAG repeats in the AR gene correlate with young age at diagnosis of prostate cancer, but not with higher risk of the disease. Selection of patients with early onset prostate cancer in case–control studies could therefore lead to an over-estimation of the risk of prostate cancer for men with short CAG repeats. An association between long CAG repeats and good response to endocrine therapy was also found, but the mechanism and clinical relevance are unclear. © 1999 Cancer Research Campaign

**Keywords:** prostatic neoplasms; cancer risk; androgen receptor; genetics; epidemiology

Family history is the best characterized risk factor for prostate cancer together with age and race (Dijkman and Debruyne, 1996). In many families, aggregation of prostate cancer is caused by autosomal dominantly inherited susceptibility genes with high penetrance (Carter et al, 1992). One of these susceptibility genes is located on chromosome 1q24–25 (Smith et al, 1996). Epidemiological studies have shown that the risk of prostate cancer is increased two- to threefold for male first-degree relatives of men with prostate cancer. Many of them have indicated that the risk may be higher for brothers than for sons of men with prostate cancer (Hayes et al, 1995; Keetch et al, 1995; Monroe et al, 1995; Narod et al, 1995; Whittemore et al, 1995; Lesko et al, 1996; Schaid et al, 1998; Bratt et al, 1999). These findings led to the hypothesis that prostate cancer susceptibility may be transmitted by X-linked or recessive inheritance in some families (Monroe et al, 1995), and recently a prostate cancer susceptibility gene was located to the long arm of the X chromosome (Xu et al, 1998).

Another gene on the X chromosome which may be involved in hereditary prostate cancer susceptibility is the androgen receptor (AR) gene. Androgens are potent growth factors for the normal prostatic gland and are considered as important promoting factors for the development of prostatic adenocarcinoma (Hakimi et al, 1996). Circulating androgens diffuse into prostatic cells where they bind to the AR. The androgen–AR complex is transferred to the cell nucleus where it binds to DNA and influences the transcription of numerous genes (the so-called transactivation function of the AR). The N-terminal transactivation domain of the AR contains several polymorphic amino acid repeats, the most variable of which is a glutamine repeat. This glutamine repeat is encoded by a CAG repeat in exon 1 of the AR gene. About 75% of whites have between 19 and 25 repeats (Giovannucci et al, 1997). There is an inverse linear relation between CAG repeat length and AR transactivation function (Chamberlain et al, 1994; Kazemi-Esfarjani et al, 1995; Tut et al, 1997), in the upper extreme end (> 40 repeats) manifested as the rare Kennedy's disease with symptoms such as muscular atrophy, low virilization and subfertility (La Spada et al, 1991). Prostatic cells with short CAG repeats in the AR gene would consequently have increased sensitivity to proliferative stimulation of androgens, and the conditions for malignant growth would therefore be better (Hakimi et al, 1996). Interestingly, the frequency distribution of AR CAG repeat length in different races co-varies with prostate cancer incidence (Irvine et al, 1995). African-American men, who have the highest incidence of prostate cancer in the world, more frequently have short CAG repeats than white and Asian-Americans (Irvine et al, 1995). Further indications of a relationship between the number of CAG repeats in the AR and prostate cancer risk were found in case–control studies, in which AR genes with short repeats were

*Received 2 February 1999*
*Accepted 20 April 1999*

*Correspondence to:* O Bratt more common among men with prostate cancer than among controls (Giovannucci et al, 1997; Hakimi et al, 1997; Ingles et al, 1997).

We have previously reported on a population-based case–control study of family history as a risk factor for prostate cancer (Bratt et al, 1999). That study showed, in concordance with most other case–control studies, a larger risk of prostate cancer for brothers (relative risk 3.6) than for sons (relative risk 2.2) of men with prostate cancer. This led us to consider the possibility of an X-linked susceptibility for prostate cancer in this population. Short CAG repeats in the AR was a possible candidate for such an X-linked susceptibility, and we did therefore perform a case–control study to test this hypothesis. We also investigated the relations between CAG repeat length and tumour stage and grade, age at diagnosis and response to endocrine therapy.

### MATERIALS AND METHODS

### Study population

Patients with a histopathological diagnosis of prostatic adenocarcinoma were selected from participants in a study of family history as a risk factor for prostate cancer (Bratt et al, 1999).

Their family history of prostate cancer was assessed with questionnaires. The diagnoses of relatives reported to have prostate cancer were verified in the regional tumour registry. The present study included 92 cases with a negative family history of prostate cancer, referred to as sporadic cases in the following text, 68 cases reporting a father ($n$ = 27) or a brother ($n$ = 41) with prostate cancer (later referred to as familial cases), and 30 patients with a pedigree consistent with autosomally dominant hereditary prostate cancer (Carter et al, 1993). All 190 prostate cancer patients were whites living in southern Sweden at the time of diagnosis. Age, tumour stage (localized, locally advanced, or metastatic), WHO tumour grade (low, intermediate, or high), and prostate-specific antigen (PSA) level at the time of diagnosis were registered for all cases. Tumour grade and stage had similar distributions in the three groups, whereas age at diagnosis had not: the median age was 71 years for sporadic cases (range 45–89 years), 69 years for familial cases (range 47–83 years) and 65 years for hereditary cases (range 44–78 years).

Ninety-one of the prostate cancer patients received endocrine therapy: 78 were treated with gonadotropin-releasing hormone analogues, 12 with bilateral orchidectomy, and one received bicalutamide as monotherapy. No-one was treated with maximal androgen blockade. The response to endocrine therapy was categorized as good, intermediate, or poor. The categories for patients with skeletal metastases were defined as follows: good response was defined as relief of symptoms with a duration of 2 years or more; intermediate response as initial relief of symptoms followed by a symptomatic progress after 1–2 years; and poor response as symptomatic progress within 1 year from start of treatment. For patients without skeletal metastases good response was defined as normalization of PSA (< 4 ng $ml^{-1}$), relief of symptoms and absence of any kind of progress for more than 2 years; the response was categorized as poor when PSA did not decrease to normal or if the disease progressed biochemically (PSA > 4 ng $ml^{-1}$) or clinically within 1 year from start of therapy; responses falling in-between these categories were considered as intermediate. Follow-up sufficient to categorize the response to endocrine therapy was available for 73 patients.

The control group for calculation of prostate cancer risk consisted of 186 AR alleles from 93 white women without any history of cancer, living in the same geographical area as the prostate cancer patients.

### Analysis of CAG repeat length in the AR gene

Genomic DNA from leukocytes was used as template in polymerase chain reaction (PCR) amplification of an approximately 150 bp region in exon 1 of the AR gene comprising the polymorphic CAG repeat. Primer sequences were GCGCGAAGT-GATCCAGAA (forward) and GTTGCTGTTCCTCATCCA (reverse) (Lubahn et al, 1989). The forward primer was labelled by T4 polynucleotide kinase and $^{32}$P-ATP, and PCR products were analysed by electrophoresis in denaturing 6% polyacrylamide gels. Alternatively, PCR products were fluorescence labelled by a TET-tagged forward primer and analysed in an ABI310 DNA fragment analyser using POP4 polymer and Genescan software (Perkin-Elmer). In either case, three different PCR products containing a sequence verified number of CAG repeats (14, 21 and 31 repeats) were used as standards for calculation of the numbers of repeats in our samples.

### Statistics

Unconditional logistic regression was used to analyse the relation between CAG repeat length and risk of prostate cancer. CAG repeat length was analysed both as a discrete numerical variable and categorized in tertiles. Hereditary prostate cancer is considered to be caused by germline mutations in specific dominant susceptibility genes with high penetrance (Carter et al, 1993), and the number of CAG repeats is therefore unlikely to contribute significantly to prostate cancer risk in this group of patients. Hence, the 30 cases with hereditary prostate cancer were excluded in these analyses.

The relations between CAG repeat length and tumour stage, tumour grade, and response to endocrine therapy were assessed with one-way analysis of variance and logistic regression, whereas the association between repeat length and age at diagnosis was analysed with univariate and multivariate linear regression and by calculating Pearson’s correlation coefficient. All significance tests were two-tailed.

### RESULTS

Mean CAG repeat length was 21.8 (range 15–31) for prostate cancer cases and 21.7 (range 12–30) in the control group. Mean repeat length was 22.1 for patients with sporadic prostate cancer, 22.3 for patients with a brother diagnosed with prostate cancer, 20.9 for patients whose father had prostate cancer and 21.0 for patients with hereditary prostate cancer. The differences in repeat length between these four groups were not statistically significant. The study included samples from nine brother pairs with prostate cancer. CAG repeat length was not significantly different for the five brother pairs in which CAG repeat length was equal for both brothers (mean 22.8), compared to the four brother pairs with unequal repeat length (mean 20.7).

The distribution of CAG alleles was similar for cases and controls (Figure 1). Logistic regression analysis showed no significant association between CAG repeat length and risk of prostate cancer, neither when CAG repeat length was categorized

*© 1999 Cancer Research Campaign*

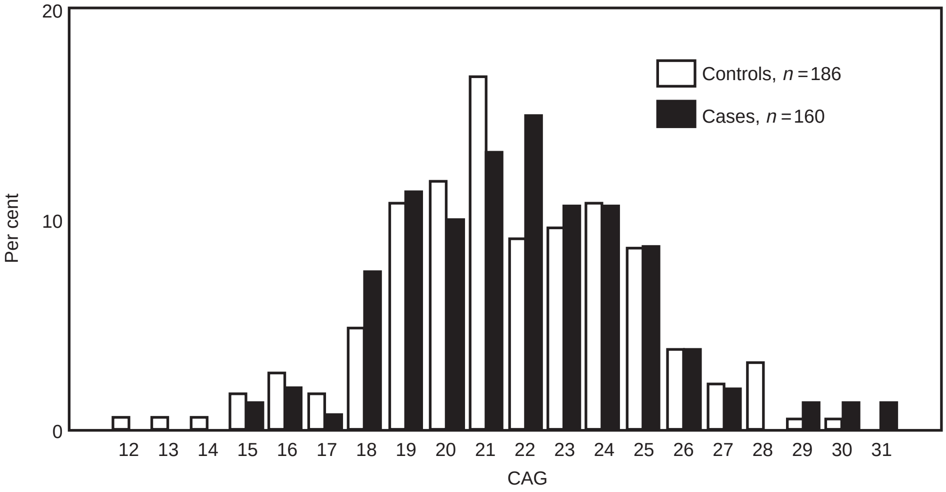

**Figure 1** Frequency distribution of CAG repeat length in the AR gene from 160 men with non-hereditary prostate cancer and 186 AR gene alleles from the population

**Table 1** Relation between CAG repeat length in the AR gene and age at diagnosis of non-hereditary prostate cancer

| Age at diagnosis (years) | Mean CAG repeats (No.) | Cases (No.) |
|---|---|---|
| <65 | 21.0 | 44 |
| 65–75 | 22.2 | 79 |
| >75 | 22.4 | 37 |
| **CAG repeats (No.)** | **Mean age at diagnosis (years)** | **Cases (No.)** |
| 15–20 | 67.3 | 52 |
| 21–23 | 68.7 | 62 |
| 24–31 | 70.9 | 46 |

**Table 2** Odds ratios for good response to endocrine therapy in 73 patients with prostate cancer, calculated with multiple backwards logistic regression

| Variable | Odds ratio | 95% CI | *P*-value |
|---|---|---|---|
| CAG repeat length | 1.25 | 1.00–1.57 | 0.05 |
| Tumour grade | 0.38 | 0.13–0.84 | 0.02 |
| Log PSA | 0.34 | 0.12–0.94 | 0.04 |
| Tumour stage | 0.69 | 0.23–2.09 | 0.51 |

CAG repeat length was analysed as a discrete numerical variable and odds ratios were calculated for increments of one CAG triplet. Tumour stage was excluded from the final equation since it was not significantly related to response. CI – confidence interval, Log PSA – the logarithm of the pre-therapeutic level of prostatic-specific antigen.

in tertiles (data not shown), nor when it was analysed as a discrete numerical variable. Odds ratios calculated for a decrement of one CAG triplet were 0.97 (95% confidence interval (CI) 0.91–1.04, $P$ = 0.47) for non-hereditary prostate cancer, 1.01 (95% CI 0.93–1.10, $P$ = 0.75) for high-grade or high-stage non-hereditary prostate cancer, and 1.08 (95% CI 0.97–1.20, $P$ = 0.15) for non-hereditary prostate cancer diagnosed before the age of 65 years. The odds ratios were not higher when only cases with a family history consistent with X-linked prostate cancer susceptibility were included (data not shown).

Patients with high-grade tumours had shorter CAG repeats (mean 21.0) than patients with low or intermediate grade tumours (mean 22.0), but the difference was not statistically significant ($P$ = 0.07). The odds ratio for high-grade tumours was 1.13 per each CAG triplet decrement (95% CI 0.99–1.29, $P$ = 0.07). The relation between CAG repeat length and tumour stage was similar: patients with locally advanced or metastatic disease had shorter CAG repeats (mean 21.4) than patients with localized disease (mean 22.2, $P$ = 0.07). The odds ratio for locally advanced or metastatic disease was 1.09 (95% CI 0.99–1.21, $P$ = 0.08).

CAG repeat length did significantly correlate with age at diagnosis of prostate cancer (correlation coefficient 0.16, $P$ = 0.03). Univariate regression analysis showed a significant linear relation between CAG repeat length and age at diagnosis for sporadic and familial cases (regression coefficient 0.48, $P$ = 0.03), but not for hereditary cases (regression coefficient 0.15, $P$ = 0.8). The relation between CAG repeat length and age was similar for familial cases having a father with prostate cancer and for those having a brother with prostate cancer. The regression coefficient and $P$-value did not change when a multiple regression analysis was performed adding tumour grade and stage as co-variates. Mean CAG repeat length in different age groups and mean age at diagnosis for cases grouped in tertiles of CAG repeat length are shown in Table 1.

We also found a significant association between CAG repeat length and response to endocrine therapy (variance analysis, $P$ = 0.04). The 45 patients with good response had longer CAG repeats (mean 22.7) than the 11 with intermediate response (mean 20.8) and the 17 with poor response (mean 21.1). In univariate logistic regression the odds ratio for good response was 1.26 (95% CI 1.05–1.52, $P$ = 0.01) per each CAG triplet increment. When adjusting for tumour grade, tumour stage and PSA level at initiation of therapy in a multiple backward logistic regression model, CAG repeat length remained a significant independent predictor of response. This means that a good response to endocrine therapy was four times more likely for patients with 25 repeats than for patients with 19 repeats (a 6-CAG triplets increment, $1.26^6 = 4.0$), which represents the upper and lower limits of the 75th percentile of the distribution of CAG repeat length in a white population (Giovannucci, 1997). That tumour stage was not significantly associated with response to endocrine therapy in multivariate

 © 1999 Cancer Research Campaign analysis is explained by that the categories for response were different for patients with non-metastatic disease than for patients with metastasis.

### DISCUSSION

The present study gave no evidence of an association between short CAG repeats in exon 1 of the AR gene and increased risk of prostate cancer. The frequency distribution of CAG repeat length was strikingly similar for cases and controls, with no CAG repeat interval more common among cases than among controls (Figure 1). We did, however, in accordance with Hardy et al (1996), find a significant correlation between CAG repeat length and age at diagnosis of sporadic and familial prostate cancer. Assuming that prostatic cells with short glutamine repeats in the AR are more sensitive to circulating androgens (Chamberlain et al, 1994; Kazemi-Esfarjani et al, 1995; Tut et al, 1997), a correlation between short CAG repeats in the AR gene and low age at diagnosis of prostate cancer is to be expected: prostatic adenocarcinoma cells with short CAG repeats would be more proliferative and therefore progress faster from microscopic lesions to clinically overt tumours.

If CAG repeat length correlates with age at onset of prostate cancer, but not with risk of prostate cancer per se, age at onset is a confounder in case–control studies of CAG repeat length and prostate cancer risk. If predominantly early onset cases are included, data are not valid for direct calculation of prostate cancer risk, which would be over-estimated if the effect of age at diagnosis of cases is not adjusted for. Two of four previous case–control studies included mainly prostate cancer cases diagnosed before the age of 65 (Ingles et al, 1997; Stanford et al, 1997), whereas age at diagnosis was not reported in the other two studies (Giovannucci et al, 1997; Hakimi et al, 1997). Our study included patients of all ages, but when the analysis was restricted to cases diagnosed before the age of 65, the odds ratio for prostate cancer increased to 1.08 per each CAG triplet decrement. Consequently, the odds ratio was 1.6 ($1.08^6$) per six CAG triplets decrement, which is in the same range as previously reported for six CAG decrements (Giovannucci et al, 1997).

We found no association between CAG repeat length and age at diagnosis for patients with hereditary prostate cancer, which may reflect a pathogenetical difference between prostatic tumours caused by germline mutations in specific prostate cancer susceptibility genes and non-hereditary prostate cancer.

Many epidemiological studies have shown a higher risk of prostate cancer for brothers than for sons of men with prostate cancer (Hayes et al, 1995; Keetch et al, 1995; Monroe et al, 1995; Narod et al, 1995; Whittemore et al, 1995; Lesko et al, 1996; Schaid et al, 1998; Bratt et al, 1999). These findings led to the hypothesis that prostate cancer susceptibility may be X-linked in some families (Monroe et al, 1995), and were the incitement for the present study. However, our results were totally negative as regards an association between CAG repeat length and prostate cancer risk. Information bias and detection bias likely contribute to the above mentioned findings in the epidemiological studies (Bratt et al, 1999), but recently a prostate cancer susceptibility gene was indeed mapped to the long arm of the X chromosome (Xu et al, 1998). This gene, named *HPCX*, is most likely a more important cause of X-linked inheritance of prostate cancer, than is CAG repeat length polymorphism in the AR gene. There are, however, other polymorphisms in the AR gene that might be of importance for prostate cancer pathogenesis (Irvine et al, 1995; Ross et al, 1998).

The cause of the association between long CAG repeats and good response to endocrine therapy is obscure. Circulating androgenic hormones stimulate proliferation of most prostatic adenocarcinomas, and androgen withdrawal is standard therapy for patients with advanced prostate cancer. As a rule, the disease will eventually become androgen-independent and escape therapeutic control, though the duration of response to androgen withdrawal therapy varies widely. The effects of androgens on prostatic carcinoma cells are mediated by the AR, and inter-individual differences in AR activity may thus contribute to the variable clinical response to endocrine therapy.

Loss of hormonal dependence is commonly caused by development of less differentiated tumour clones through accumulative genetic abnormalities occurring mainly during cell division (Rinker-Schaeffer et al, 1994). Assuming that prostate cancer cells with short CAG repeats in the AR divide more rapidly, they would be more likely to give origin to androgen-independent cell clones early in tumour progression. If this hypothesis were true, one would expect an association between short CAG repeats and high tumour grade. Such an association was indeed found by Giovannucci et al (1997), and there was a trend in that direction in the present study. High-grade tumours are somewhat more common in men with early onset prostate cancer (Grönberg et al, 1994). Short CAG repeats might be a common risk factor for early onset disease and high-grade tumours and could thus contribute to this relation, but other explanations are possible (Bratt et al, 1998).

Hardy and co-workers (Hardy et al, 1996) found no relation between CAG repeat length and response to endocrine therapy. Their classification of response and statistical work-up were entirely different from the evaluation in our study and comparisons between their study and ours are therefore difficult to make. The number of patients in our study was small and the time of follow-up was short, and further studies are needed before any conclusions can be made regarding a possible causative relation between CAG repeat length and response to endocrine therapy.

We conclude that in the studied population of whites in southern Sweden, short CAG repeats in the AR gene correlate with younger age at diagnosis of sporadic and familial prostate cancer, but not with increased risk of prostate cancer in general. An association between long CAG repeats and good response to endocrine therapy was also found, but the mechanism and clinical relevance are unclear. Since CAG repeat length correlates with age at onset of prostate cancer, selection of patients with early onset disease is a potential confounder in case–control studies of CAG repeat length and prostate cancer risk. If mainly early onset cases are included, the general risk of prostate cancer for men with short CAG repeats is over-estimated if the effect of age at diagnosis is not adjusted for. Thus, the present knowledge about how CAG repeat length in the AR gene is related to prostate cancer risk and biology is still very limited.

### ACKNOWLEDGEMENTS

This study was supported by grants from AB Leo Research Foundation, Pharmacia-Upjohn, Tatjana och Jacob Kamras' Forskningsfond, Gunnar, Arvid och Elisabeth Nilssons stiftelse för bekämpning av cancersjukdomar, and Crafoordska Stiftelsen.

© 1999 Cancer Research Campaign 

### REFERENCES

Bratt O, Kristoffersson U, Lundgren R and Olsson H (1999) Familial and hereditary prostate cancer in southern Sweden. A population-based case–control study. *Eur J Cancer* **35**: 272–277

Bratt O, Kristoffersson U, Olsson H and Lundgren R (1998) Clinical course of early onset prostate cancer with special reference to family history as a prognostic factor. *Eur Urol* **34**: 19–24

Carter BS, Beaty TH, Steinberg GD, Childs B and Walsh PC (1992) Mendelian inheritance of familial prostate cancer. *Proc Natl Acad Sci USA* **89**: 3367–3371

Carter BS, Bova G, Beaty TH, Steinberg GD, Childs B, Isaacs W and Walsh PC (1993) Hereditary prostate cancer: epidemiologic and clinical features. *J Urol* **150**: 797–802

Chamberlain N, Driver E and Miesfeld R (1994) The length and location of CAG trinucleotide repeats in the androgen receptor N-terminal domain affect transactivation function. *Nucleic Acid Res* **22**: 3181–3186

Dijkman G and Debruyne F (1996) Epidemiology of prostate cancer. *Eur Urol* **30**: 281–295

Giovannucci E, Stampfer M, Krithivas K, Brown M, Brufsky A, Talcott J, Hennekens C and Kantoff P (1997) The CAG repeat within the androgen receptor gene and its relationship to prostate cancer. *Proc Natl Acad Sci USA* **94**: 3320–3323

Grönberg H, Damber J-E, Jonsson H and Lenner P (1994) Patient age as a prognostic factor in prostate cancer. *J Urol* **152**: 892–895

Hakimi J, Rondinelli R, Schoenberg M and Barrack E (1996) Andogen-receptor gene structure and function in prostate cancer. *World J Urol* **14**: 329–337

Hakimi J, Schoenberg M, Rondinelli R, Piantadosi S and Barrack E (1997) Androgen receptor variants with short glutamine or glycine repeats may identify unique subpopulations of men with prostate cancer. *Clin Cancer Res* **3**: 1599–1608

Hardy D, Scher H, Bogenreider T, Sabbatini P, Zhang Z-F, Nanus D and Catterall J (1996) Androgen receptor CAG repeat lengths in prostate cancer: correlation with age of onset. *J Clin Endocrinol Metab* **81**: 4400–4405

Hayes RB, Liff JM, Pottern LM, Greenberg RS, Schoenberg JB, Schwartz AG, Swanson GM, Silverman DT, Morris Brown L, Hoover RN and Fraumeni JF (1995) Prostate cancer risk in U.S blacks and whites with a family history of cancer. *Int J Cancer* **60**: 361–364

Ingles S, Ross R, Yu M, Irvine R, La Pera G, Haile R and Coetzee G (1997) Association of prostate cancer risk with genetic polymorphisms in vitamin D receptor and androgen receptor. *J Natl Cancer Inst* **89**: 166–170

Irvine R, Yu M, Ross R and Coetzee G (1995) The CAG and GGC microsatellites of the androgen receptor gene are in linkage disequilibrium in men with prostate cancer. *Cancer Res* **55**: 1937–1940

Kazemi-Esfarjani P, Trifiro M and Pinsky L (1995) Evidence for a repressive function of the long polyglutamine tract in the human androgen receptor: possible pathogenetic relevance for the (CAG)n-expanded neuronopathies. *Hum Mol Genet* **4**: 523–527

Keetch DW, Rice JP, Suarez BK and Catalona WJ (1995) Familial aspects of prostate cancer: a case control study. *J Urol* **154**: 2100–2102

La Spada A, Wilson E, Lubahn D, Harding A and Fischbeck K (1991) Androgen receptor gene mutations in X-linked spinal and bulbar muscular dystrophy. *Nature* **352**: 77–79

Lesko S, Rosenberg L and Shapiro S (1996) Family history and prostate cancer risk. *Am J Epidemiol* **144**: 1041–1047

Lubahn D, Brown T, Simental J, Higgs H, Migeon C, Wilson E and French F (1989) Sequence of the intron/exon junctions of the coding region of the human androgen receptor gene and identification of a point mutation in a family with complete androgen insensitivity. *Proc Natl Acad Sci USA* **86**: 9534–9538

Monroe K, Yu M, Kolonel L, Coetzee G, Wilkens L, Ross, RK and Henderson B (1995) Evidence of an X-linked or recessive genetic component to prostate cancer risk. *Nature Med* **1**: 827–829

Narod S, Dupont A, Cusan L, Diamond P, Gomez J-L, Suburu R and Labrie F (1995) The impact of family history on early detection of prostate cancer (letter). *Nature Med* **1**: 99–101

Rinker-Schaeffer C, Partin A, Isaacs W, Coffey D and Isaacs J (1994) Molecular and cellular changes associated with the acquisition of metastatic ability by prostatic cancer cells. *Prostate* **25**: 249–265

Ross R, Pike M, Coetzee G, Reichardt J, Yu M, Feigelson H, Stanczyk F, Kolonel L and Henderson B (1998) Androgen metabolism and prostate cancer: establishing a model of genetic susceptibility. *Cancer Res* **58**: 4497–4504

Schaid D, McDonnel S, Blute M and Thibodeau S (1998) Evidence for autosomal dominant inheritance of prostate cancer. *Am J Hum Genet* **62**: 1425–1438

Smith JR, Freije D, Carpten JD, Grönberg H, Xu J, Isaacs SD et al (1996) Major susceptibility locus for prostate cancer on chromosome 1 suggested by a genome-wide search. *Science* **274**: 1371–1374

Stanford J, Just J, Gibbs M, Wicklund K, Neal C, Blumenstein B and Ostrander E (1997) Polymorphic repeats in the androgen receptor gene: molecular markers of prostate cancer risk. *Cancer Res* **57**: 1194–1198

Tut T, Ghadeesy F, Trifiro M, Pinsky L and Yong E (1997) Long polyglutamine tracts in the androgen receptor are associated with reduced trans-activation, impaired sperm production, and male infertility. *J Clin Endocrinol Metab* **82**: 3777–3782

Whittemore AS, Wu AH, Kolonel LN, John EM, Gallagher RP, Howe GR, West DW, Teh C-Z and Stamey T (1995) Family history and prostate cancer risk in black, white, and Asian men in the United States and Canada. *Am J Epidemiol* **141**: 732–740

Xu J, Meyers D, Freije D, Isaacs S, Wiley K, et al (1998) Evidence for a prostate cancer susceptibility locus on the X chromosome (letter). *Nature Genetics* **20**: 175–179

*© 1999 Cancer Research Campaign*
